# Activation of Yes-Associated Protein/PDZ-Binding Motif Pathway Contributes to Endothelial Dysfunction and Vascular Inflammation in AngiotensinII Hypertension

**DOI:** 10.3389/fphys.2021.732084

**Published:** 2021-09-28

**Authors:** Qian Xu, Kunping Zhuo, Ruiping Cai, Xiaomin Su, Lu Zhang, Yueyang Liu, Lin Zhu, Fu Ren, Ming-Sheng Zhou

**Affiliations:** ^1^Department of Physiology, Shenyang Medical College, Shenyang, China; ^2^Department of Pharmacology, Shenyang Pharmaceutical University, Shenyang, China; ^3^Department of Biochemistry and Molecular Biology, Shenyang Medical College, Shenyang, China; ^4^Department of Anatomy, Shenyang Medical College, Shenyang, China

**Keywords:** endothelial dysfunction, Hippo/YAP pathway, angiotensinII, hypertension, vascular inflammation

## Abstract

Yes-associated protein (YAP) and its associated coactivator of PDZ-binding motif (TAZ) are co-transcriptional regulators and down effectors of the Hippo signaling pathway. Recent studies have shown that the Hippo/YAP signaling pathway may play a role in mediating vascular homeostasis. This study investigated the role of YAP/TAZ in endothelial dysfunction and vascular inflammation in angiotensin (Ang)II hypertensive mice. The infusion of AngII (1.1 mg/kg/day by mini-pump) for 3 weeks induced the activation of YAP/TAZ, manifested by decreased cytosolic phosphor-YAP and phosphor-TAZ, and increased YAP/TAZ nuclear translocation, which were prevented by YAP/TAZ inhibitor verteporfin. AngII significantly increased systolic blood pressure (SBP), macrophage infiltration, and expressions of proinflammatory cytokines, and impaired endothelial function in the aorta of the mice. Treatment with verteporfin improved endothelial function and reduced vascular inflammation with a mild reduction in SBP. AngII also induced YAP/TAZ activation in human umbilical vein endothelial cells *in vitro*, which were prevented by LB-100, an inhibitor of protein phosphatase 2A (PP2A, a major dephosphorylase). Treatment with LB-100 reversed AngII-induced proinflammatory cytokine expression and impairment of phosphor-eNOS expression *in vitro*. Our results suggest that AngII induces YAP/TAZ activation *via* PP2A-dependent dephosphorylation, which may contribute to the impairment of endothelial function and the induction of vascular inflammation in hypertension. YAP/TAZ may be a new target for hypertensive vascular injury.

## Introduction

Hypertension is a major risk factor for the development of cardiovascular diseases (CVDs). Long-term high blood pressure causes vascular injury, which is associated with endothelial dysfunction and increased arterial stiffness and thickness (Ponticos and Smith, 2014). Despite extensive studies, the mechanisms of hypertension-induced vascular injury are not fully understood. It is well-known that high blood pressure has hemodynamic derangement, such as increased hemodynamic stress and blood flow turbulence. Turbulent blood flow can activate many signaling pathways, such as increased inflammatory cytokine expression and reactive oxygen species (ROS) production in the endothelium (Masi et al., 2019).

Yes-associated protein (YAP) is an essential signaling molecule to sense biomechanical force, including hemodynamic stress and extracellular stiffness. YAP and its associated coactivator of PDZ-binding motif (TAZ) are the critical downstream effectors of the Hippo pathway, and YAP/TAZ interacts with TEAD/TEF family of transcription factors and promotes cell proliferation and survival (Panciera et al., 2017; Ando et al., 2018). It has been shown that the Hippo/YAP pathway can be activated by sensing extracellular stiffness and play an important role in the regulation of cell proliferation, apoptosis, and differentiation (Moya and Halder, 2019; Rausch and Hansen, 2020). The dysregulation of the Hippo/YAP pathway may lead to uncontrolled cell proliferation and promote human cancers (Moroishi et al., 2015). Recent studies suggested that the Hippo/YAP pathway may play a role in the pathogenesis of CVDs (Zhou and Zhao, 2018; Yu et al., 2020). Wang et al. have reported that vascular endothelial YAP/TAZ can be activated by atherosclerotic turbulent flow. The activation of YAP/TAZ promotes vascular inflammation and atherogenesis (Wang L. et al., 2016; Ranchoux et al., 2018; Wang and Zhang, 2020).

The activation of the renin-angiotensin system (RAS) has been implicated in hypertension and hypertension-associated vascular complication (Schulman et al., 2006). Angiotensin (Ang)II is a proinflammatory mediator. The infusion of AngII into the mice causes endothelial dysfunction, vascular inflammation, and injury. Recent studies suggest that AngII may involve the regulation of the Hippo/YAP pathway (Wennmann et al., 2014). Wennmann et al. have shown that AngII can increase unphosphorylated YAP aggregation to induce YAP activation by downregulating the expression of large tumor suppressor kinase (LATS)1/2 kinase (an upstream molecule of YAP) in HEK293T cells *in vitro* (Wennmann et al., 2014) and increase YAP expression in the media of carotid arteries of the rats *in vivo* (Lin et al., 2018).

It is generally accepted that hypertension is a chronic vascular inflammatory disease associated with endothelial activation and dysfunction (Guzik and Touyz, 2017). Endothelial cells are directly exposed and serve as a sensor to blood shear stress, as disturbed blood flow may activate YAP to promote endothelial activation and dysfunction in hypertension. In this study, we used YAP activation inhibitor verteporfin to treat AngII hypertensive mice for 3 weeks. The results reported, in this study, demonstrate that verteporfin inhibits AngII-induced YAP/TAZ activation *in vivo* and *in vitro*, reduces vascular inflammation, and improves endothelial dysfunction in AngII hypertensive mice *in vivo.* Our results suggest that the Hippo/YAP pathway may play a role in AngII-induced vascular injury.

## Materials and Methods

### Animals and Experimental Protocols

Six-week-old male C57BL/6 mice were purchased from the Beijing Charles River Animal Laboratory (Beijing, China). All animal experiments complied with the international standards stated in the Guide for the Care and Use of Laboratory Animals. All animal protocols were approved by the Institutional Animal Care and Use Committee of Shenyang Medical College (approved animal protocol #: 17-25). The mice were randomly divided into the following three groups and treated for 3 weeks: (1) control (Ctr) group: the mice underwent sham procedures with the implantation of an empty osmotic mini-pump (*n* = 6); (2) AngII hypertensive group (AngII): the mice were implanted with an osmotic mini-pump (Alzet model 1002D, DURECT Inco., Cupertino, CA, United States) with the infusion of AngII (1.1 mg/kg/day, Sigma Aldrich, St. Louis, MO, United States, *n* = 6); (3) AngII with verteporfin treatment (AngII/Ve): the mice were implanted with an osmotic mini-pump with AngII plus verteporfin (Selleck, Houston, TX, United States) treatment (*n* = 6). We have previously shown that the mice that received the pressor dose of AngII for 5 days can develop hypertension and cardiovascular injury (Zhang et al., 2021). Verteporfin was dissolved in 10% of dimethylsulfoxide (DMSO) saline solution and administrated at a dosage of 60 mg/kg into mice by intraperitoneal injection every other day. The mice in Ctr and AngII groups were administrated with an intraperitoneal injection of the same amount (0.1 ml) of 10% of DMSO solvent every other day to eliminate the possible effects of this solvent in the mice. Verteporfin is an inhibitor of the YAP-TEAD complex and has been shown to inhibit the translocation of YAP/TAZ into the nuclei and decrease the protein expression of YAP/TAZ (Szeto et al., 2016; Wei et al., 2017). The systolic blood pressure (SBP) and diastolic blood pressure (DBP) were measured by using the tail-cuff method (Softron Biotechnology Co., Ltd., Beijing, China) in the conscious mice as previously described (Huang et al., 2018). The mice were trained daily for 5 consecutive days before the beginning of the experiment. Blood pressure was measured at baseline (before mini-pump implantation) and thereafter once a week. At least five successive readings were recorded and averaged for each mouse. The mice were euthanized by an overdose of anesthesia (5% chloral hydrate, administrated at a dosage of 0.4 ml/100 g body weight, I.P.). The aorta was harvested and weighted.

### Histological Analysis

The thoracic aorta (1 cm below the highest point of the aortic arch) was isolated and fixed in 4% of paraformaldehyde in phosphate-buffered saline. The tissue samples were cut into 4 μm-thick sections and stained with H&E (Sigma Aldrich, St. Louis, MO, United States). The slides were photographed using a Leica DM4B fluorescence microscope. Four images in four non-consecutive slides per animal were acquired and analyzed using the Image J 1.48V software system, and the radial thickness of the media was measured. Trichrome staining (Masson, Sigma Aldrich, St. Louis, MO, United States) was used to assess aortic fibrosis. The semiquantitative analysis was performed, using Image J 1.48V software system, and aortic fibrosis was expressed as a percentage of positive staining area with the total selected area. The image quantitation and representative photomicrographs were taken in a blinded fashion that the reviewers were without the knowledge of the experimental groups.

### Organ Chamber Bath Experiments

The aorta was cleaned out of the adherent connective tissues and cut into 3-mm rings. Acetylcholine-induced endothelium-dependent vasorelaxation in aortic rings was determined by using an organ bath chamber (4-channel Tissue Bath System, DMT Inc., Denmark), as previously described (Zhou et al., 2010). Aortic rings were precontracted with 70% of norepinephrine-induced (about 30 nmol/L, Sigma Aldrich, St. Louis, MO, United States) maximal constriction, and then an accumulative concentration of acetylcholine (10^–9^ to 10^–5^ mol/L, Sigma Aldrich, St. Louis, MO, United States) was added into an organ chamber.

### Cell Culture

Human umbilical vein endothelial cells (HUVECs) were obtained from American Type Culture Collection (ATCC, Manassas, VA, United States) and cultured in a high-glucose Dulbecco’s modified Eagle’s medium (DMEM, Gibco, NY, United States), supplemented with 10% of fetal bovine serum (FBS), 100 U/ml of streptomycin, and 100 U/ml of penicillin at 37°C with 5% of CO_2_. The cells were incubated with AngII (100 nmol/L) for 24 h. In some experiments, the cells were preincubated with YAP-TAZ inhibitor verteporfin (0.5 μM) or protein phosphatase 2A C (PP2Ac) inhibitor LB-100 (0.1 μM) 1 h before AngII was applied. Veterporfin was dissolved in DMSO solution and further diluted by using the DMEM culture medium. The final concentration of DMSO is <0.01% in the cultured medium. LB100 was dissolved with the culture medium.

### Immunofluorescence and Immunohistochemistry

The aortic sections (4 μm) were cut from paraffin-embedded tissues and microwaved for 30 min at 60°C for antigen retrieval with citrate buffer (pH 6.0, ZSGB-BIO, Beijing, China). The sections were incubated with primary mouse anti-YAP [1:200 dilution with Tris-buffered saline + Tween-20 (TBST) buffer, sc-101199, Santa Cruz Biotech., Santa Cruz, CA, United States] or mouse anti-F4/80 (1:300 dilution with TBST buffer, 123101, BioLegend) overnight at 4°C, followed by the incubation with fluorescein (FITC)-conjugated goat anti-mouse secondary antibody for YAP or F4/80 (1:500 dilution with TBST buffer, Beyotime Biotechnology, Shanghai, China) at 37°C for 1 h. The nuclei were stained by counterstaining by using 4’,6-diamidino-2-phenylindole (DAPI). YAP fluorescence intensity was visualized and photographed using a Leica DM4B fluorescence microscope (Leica Microsystems Inc., Mannheim, Germany). The relative fluorescence intensity (RFI) of nuclear YAP was determined using the Image J 1.48V software system and normalized by the Ctr group. Monocytes/macrophages (F4/80 positive cells) were viewed, using a fluorescence microscope, and the number of F4/80 positive cells was counted by experienced reviewers who did not know the experimental group of the mice. The images that were acquired from the incubation with lgG without primary antibodies were the negative control. In addition, monocyte and macrophage marker 2 (MOMA2), another marker for monocyte and macrophage, was determined by immunohistochemistry. The slides were incubated with primary antibody against MOMA2 (1:100 dilution with TBST buffer, MOMA2, ab33451, Abcam, Cambridge, United Kingdom) at 4°C overnight, followed by the incubation with the horseradish peroxidase-conjugated goat anti-rat lgG secondary antibody (1:200 dilution with TBST buffer, SA00001-15, Proteintech Group, Danvers, MA, United States). A Vectastain Elite ABC Kit (Vector Laboratories, Burlingham, CA, United States) was used. At least three images per sample for immunofluorescence and immunohistochemistry were acquired, and a semiquantitative analysis was performed, using the Image J 1.48V software system. The data were expressed as a percentage of MOMA2 positive areas with total selected areas.

### Western Blot

The aorta or HUVECs were lysed in RIPA lysis buffer supplemented with 1 mmol/L of phenylmethylsulfonyl fluoride, 10 μg/ml of aprotinin, and 10 μg/ml of leupeptin at 4°C for 60 min. In some experiments, the nuclear and cytoplasmic protein fractions were separated and extracted using the nucleoprotein extraction kit (Beyotime Biotechnology, Shanghai, China) according to the protocol of the manufacture. After the homogenization, protein concentrations were measured using a BCA protein assay kit (Beyotime Biotech., Shanghai, China). Of note, 30 μg of total proteins were separated by 8 or 12% of polyacrylamide gel electrophoresis and electrophoretically transferred to nitrocellulose membrane. The membranes were incubated with blocking solution (5% of milk in TBST buffer) at room temperature for 2 h and then incubated with primary antibodies against YAP (Sc-101199, Santa Cruz Biotech., Santa Cruz, CA, United States), p-YAP (13008T, Cell Signaling, Chicago, IL, United States), TAZ (4883S, Cell Signaling, Chicago, IL, United States), p-TAZ (59971S, Cell Signaling, Chicago, IL, United States), PP2Ac (2038S, Cell Signaling, Chicago, IL, United States), p-PP2Ac (Sc-271903, Santa Cruz Biotech., Santa Cruz, CA, United States), monocyte chemoattractant protein (MCP)-1 (Sc-52701, Santa Cruz Biotech., Santa Cruz, CA, United States), tumor necrosis factor (TNF)α (Sc-52746, Santa Cruz Biotech., Santa Cruz, CA, United States), HDAC1 (Sc-81598, Santa Cruz Biotech., Santa Cruz, CA, United States), phosphor-endothelial nitric oxide synthase (p-eNOS, AF3247, Affinity, Jiangsu, China), and GAPDH (60004-1-Ig, Proteintech Group, Danvers, MA, United States) at 4°C overnight (1:500 dilution with blocking solution). The membranes were incubated with horseradish peroxidase-conjugated secondary antibody (1:5,000 dilution using blocking solution) for 2 h at room temperature. The signals of luminal chemiluminescence were detected by using an Aplegen Omega Lum G Gel Documentation System (Aplegen Inc., Pleasanton, United States) and quantified using Image J 1.48V software system. HDAC1 is used as a loading control for nuclear lysates and GAPDH as a loading control for cytosolic lysate or whole lysates. Data were normalized by the Ctr group.

### PP2Ac Activity Assay

The PP2Ac activity was measured using the V2460 kit from Promega (Madison, WI, United States) according to the instruction of the manufacturer. The cells were homogenized in 300 μl of phosphatase storage buffer at 4°C for 30 s and centrifuged at 28,000*g* at 4°C for 1 h, and 250 μl of supernatant was added to the provided spin columns to remove endogenous free phosphate. The filtrate was collected, 35 μl of the filtrate was added to the wells containing the appropriate reaction solution, and incubated for 10 min at 30°C. The reaction was stopped by adding 50 μl of molybdate dye/additive mixture. The optical density was read at 630 nm using a plate reader. The enzyme activity of PP2Ac was calculated based on a standard curve.

### Statistical Analysis

The results were expressed as mean ± SE of the mean. Statistical analyses were performed using GraphPad statistical software package, and the statistical significance of difference was determined by one-way or two-way ANOVA with Bonferroni correction for multiple comparisons. Maximal response to an agonist (Emax) and the concentration of the agonist required for a half-maximal response curve (EC50) were determined and calculated from the concentration-response curve, using the best fit to a logistic sigmoid function. The Kruskal-Wallis non-parameter analysis was used for EC50. Values were considered significant when *p* < 0.05.

## Results

### The Activation of the YAP/TAZ Pathway in the Aorta of AngII Hypertensive Mice

**FIGURE 1:**
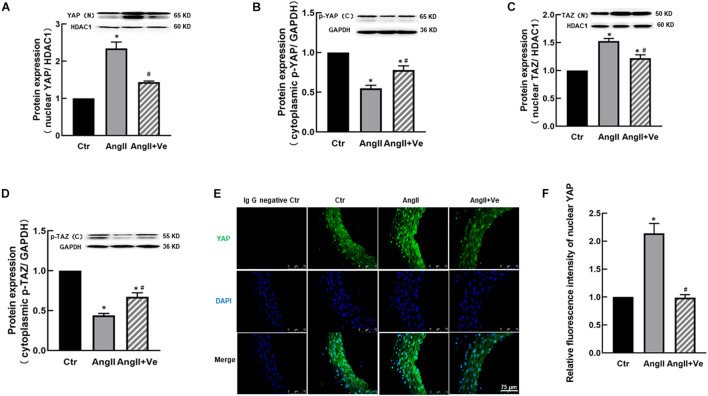
Verteporfin inhibits Yes-associated protein (YAP) and its associated coactivator of PDZ-binding motif (TAZ) activation in the aorta of angiotensin (Ang)II hypertensive mice. The protein expressions of YAP **(A)** and TAZ **(C)** in the nucleus fraction and the protein expressions of phosphor (p)-YAP **(B)** and p-TAZ **(D)** in the cytosolic fraction. **(E)** Representative immunofluorescence images of YAP (green) and nuclei were stained with DAPI (blue). **(F)** Quantitative analysis of relative immunofluorescence intensity of nuclear YAP. All values are expressed as means ± SE, *p < 0.05 vs. Ctr group. ^#^*p* < 0.05 vs. AngII group, *n* = 6.

Yes-associated protein and TAZ are major downstream effectors of the Hippo pathway, phosphorylated YAP (p-YAP) and phosphorylated TAZ (p-TAZ) are an inactive form and retained in the cytosolic apartment, and YAP/TAZ activation is initiated by dephosphorylation and promoted the relocation into the nucleus from the cytosolic apartment (Totaro et al., 2018). To determine YAP/TAZ activation, the nuclear and cytoplasmic fractions in the aortic tissues were separated. AngII increased the expressions of nuclear YAP and TAZ and decreased cytosolic p-YAP and p-TAZ expressions, and treatment with verteporfin reversed AngII-induced changes in nuclear YAP/TAZ and cytosolic p-YAP/p-TAZ expressions (Figures 1A–D). Immunofluorescence staining showed that the total immunofluorescence intensity of YAP and its colocalization with the nucleus were significantly increased in the aorta of AngII mice, which was reduced in verteporfin-treated hypertensive mice (Figures 1E,F). The results suggest that YAP/TAZ can be activated in the vasculature of AngII hypertensive mice.

### The Inhibition of YAP/TAZ With Verteporfin Lowers SBP and Improves Endothelial Function in AngII Hypertensive Mice

**FIGURE 2:**
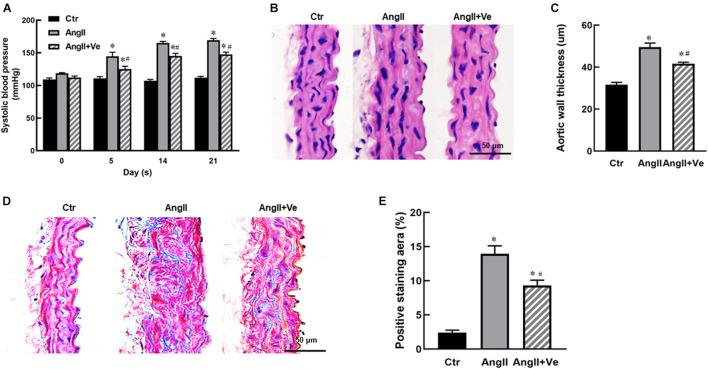
Long-term treatment with verteporfin reduces systolic blood pressure [SBP **(A)**] and aortic hypertrophy **(B,C)** and fibrosis **(D,E)** in AngII hypertensive mice. **(B)** The representative images of cross-section of the aortic wall stained with H&E, scale bar = 50 μm. **(C)** The quantitative analysis of aortic wall thickness; **(D)** the representative images of the aortic section stained with Masson-Trichrome for the evaluation of aortic fibrosis, scale bar = 50 μm. **(E)** Quantitative analysis of positive collagen-staining area (blue) in aortic section. *p < 0.05 vs. control (Ctr) group, ^#^*p* < 0.05 vs. AngII group, *n* = 6.

**FIGURE 3:**
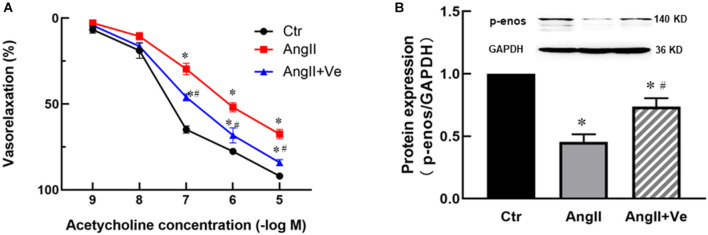
Long-term treatment with verteporfin improves endothelium-dependent relaxation to acetylcholine **(A)** and increases phosphor-endothelial nitric oxide synthase (p-eNOS) expression **(B)** in the aorta of AngII hypertensive mice. *p < 0.05 vs. Ctr group, ^#^*p* < 0.05 vs. AngII group, *n* = 6.

The infusion of AngII for 3 weeks significantly increased SBP (169 ± 7 vs. 112 ± 5 mm Hg in Ctr group) and diastolic blood pressure (DBP) in the mice, and treatment with verteporfin significantly attenuated the elevation of SBP (148 ± 8 vs. 169 ± 7 mmHg in AngII group, *p* < 0.05, Figure 2A) and DBP (Table 1) in AngII hypertensive mice. There was no significant difference in heart rate among Ctr, AngII, and AngII/Ve groups (Table 1). H&E staining showed that AngII increased the aortic thickness and hypertrophy (49.5 ± 4.9 vs. 31.7 ± 2.7 μm in Ctr group, *p* < 0.05), which was reduced in verteporfin-treated hypertensive mice (41.7 ± 1.7 vs. 49.5 ± 4.9 μm in AngII group, *p* < 0.05, Figures 2B,C). Masson-Trichrome staining revealed that AngII significantly increased positive staining areas of collagen, which were partially reduced in AngII/Ve mice (Figures 2D,E). Endothelium-dependent relaxation (EDR) to acetylcholine was significantly impaired in the aorta of AngII mice, compared with the Ctr group. The Emax was 67.5 ± 6.7% in AngII mice vs. 91.9 ± 1.2% in control mice (*p* < 0.05); EC50 was 252 ± 87 nmol/L in AngII mice vs. 39 ± 3 nmol/L in control mice (*p* < 0.05). Treatment with verteporfin significantly improved EDR to acetylcholine (Emax: 84.1 ± 4.2%, *p* < 0.05) in hypertensive mice. EC50 was 252 ± 87 nmol/L in AngII mice vs. 81 ± 14 nmol/L in control mice (*p* > 0.05). There was no significant difference in EC50 between AngII group and AngII/Ve group (Figure 3A and Table 1). Furthermore, we determined aortic p-eNOS expression, as shown in Figure 3B, and p-eNOS (ser 1177) was significantly decreased in AngII mice. Treatment with verteporfin prevented AngII-induced decrease in p-eNOS.

**TABLE 1:** Effects of verteporfin on BW, HR, DBP, and vasorelaxation in Angll hypertensive mice.

Treatment	Ctr	Angll	Angll + Ve
BW (g)	23.34 ± 0.63	24.15 ± 0.43	24.01 ± 0.47
DBP (mmHg)	65.33 ± 2.459	105.3 ± 5.402*	86.83 ± 2.272*^#^
HR	569.8 ± 28.43	511.8 ± 31.79	516.8 ± 37.84
**Vascular relaxation to acetylcholine**			
E_max_	91.94 ± 0.49	67.54 ± 2.74*	84.12 ± 1.73*^#^
EC50 (nmol/L)	39 ± 3	252 ± 87*	81 ± 14

*Data were expressed as mean + SEM; n = 6.*

*BW, body weight; HR, heart rate; DBP: diastolic blood pressure; E_max_, maximal relaxation to acetylcholine; EC50, concentration of agonist required for a half-maximal response.*

*p < 0.05, vs. Ctr group, ^#^p < 0.05, vs. Angll group.

### The Inhibition of YAP by Verteporfin Reduces Macrophage Infiltration and Vascular Inflammation in the Aorta of AngII Mice

**FIGURE 4:**
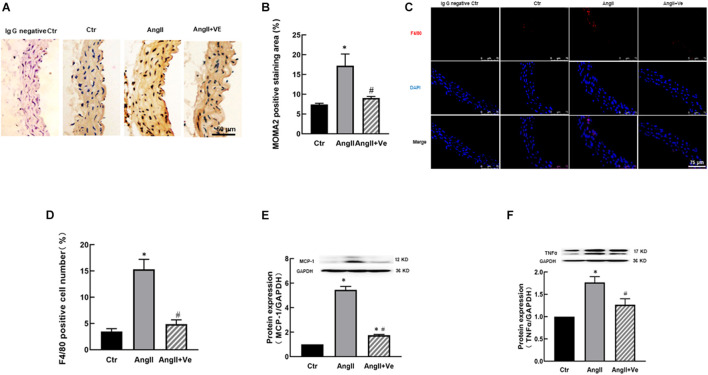
Verteporfin attenuates macrophage infiltration **(A–D)** and pro-inflammatory expressions **(E,F)** in AngII hypertensive mice. **(A)** Representative immunohistochemical images of monocyte and macrophage (MOMA)2; **(B)** the quantitative analysis of MOMA2 positive staining area; **(C)** representative immunofluorescence image of F4/80 (red), and nuclei were stained with DAPI (blue). Scale bar, 75 μm. **(D)** The number of F4/80 positive staining cells; the protein expressions of proinflammatory cytokines monocyte chemoattractant protein (MCP)-1 **(E)** and tumor necrosis factor (TNF)α **(F)**. *p < 0.05, vs. Ctr group, ^#^*p* < 0.05, vs. AngII group, *n* = 6.

AngiotensinII increases immune cell infiltration in the vascular wall, which may release proinflammatory cytokines and causes vascular inflammation (Piqueras and Sanz, 2020). We determined aortic MOMA2 expression by immunohistochemistry and F4/80 expression by immunofluorescence. As shown in Figures 4A–D, AngII increased aortic MOMA2 expression and the number of F4/80 positive cells. Treatment with verteporfin significantly reduced the MOMA2 expression and the number of F4/80 positive cells in AngII hypertensive mice. Furthermore, AngII significantly increased the expressions of proinflammatory cytokines MCP-1 and TNFα in the aorta, which were reduced in verteporfin-treated hypertensive mice (Figures 4E,F).

### AngII Activates YAP/TAZ Pathway and Increases Proinflammatory Cytokine Expression *via* Increasing PP2Ac Dephosphorylation and Activity in HUVECs

**FIGURE 5:**
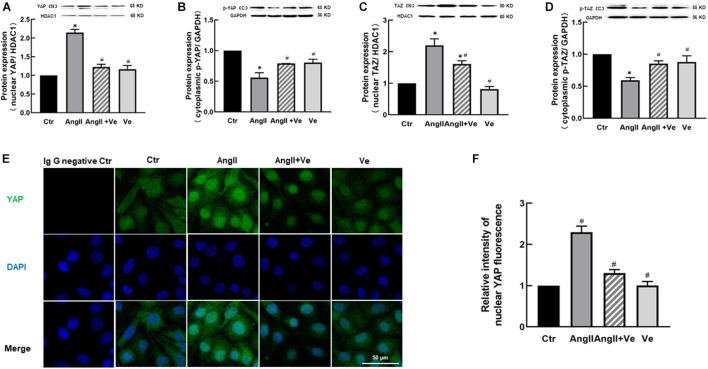
AngII induces YAP/TAZ activation in human umbilical vein endothelial cells (HUVECs). The protein expressions of YAP **(A)** and TAZ **(C)** in the nucleus fraction and the protein expressions of p-YAP **(B)** and p-TAZ **(D)** in the cytosolic fraction in HUVECs; **(E)** the representative immunofluorescence images of nucleus YAP (green), and the nuclei were stained with DAPI (blue). Scale bar, 50 μm. **(F)** Relative quantitative immunofluorescence intensity of nucleus YAP. *p < 0.05, vs. Ctr group, ^#^*p* < 0.05, vs. AngII group, *n* = 6.

**FIGURE 6:**
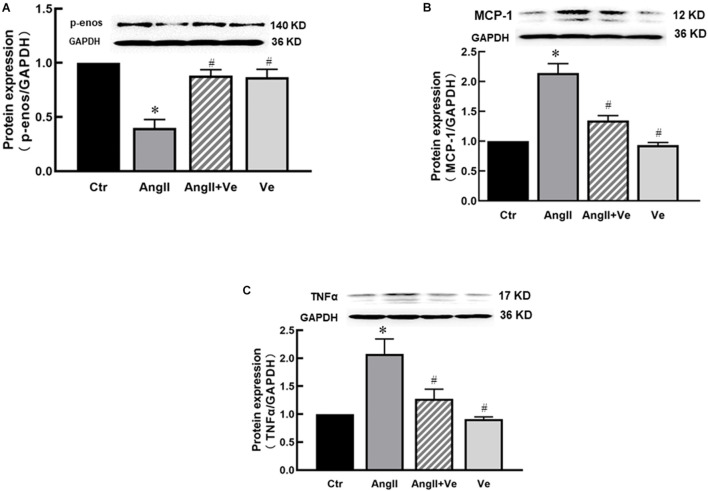
Treatment with verteporfin prevented a decrease in p-eNOS expression **(A)** and an increase in MCP-1 **(B)** and TNFα **(C)** expressions in AngII-treated HUVECs. *p < 0.05, vs. Ctr group, ^#^*p* < 0.05, vs. AngII group.

**FIGURE 7:**
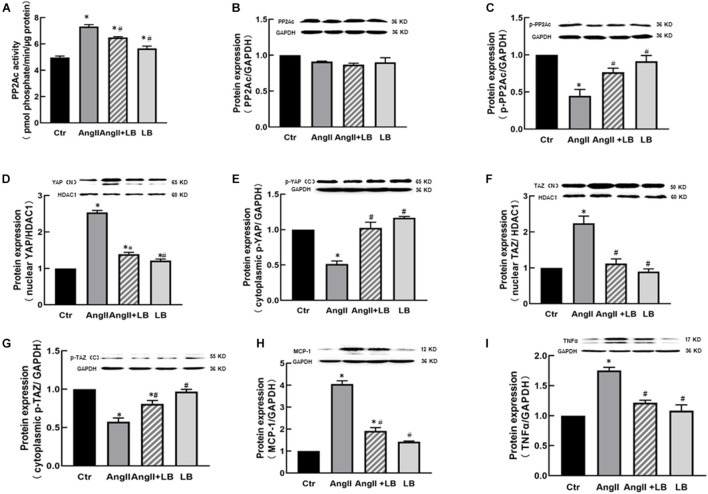
AngII induces YAP/TAZ activation and the expression of pro-inflammatory cytokines *via* protein phosphatase 2A C (PP2Ac) in HUVECs. **(A)** AngII increased PP2Ac activity; inhibition of PP2Ac with LB-100 suppressed AngII-mediated enhancement of PP2Ac activity in HUVECs. The protein expressions of PP2Ac **(B)** and p-PP2Ac **(C)**; **(D–G)** Inhibition of PP2Ac with LB-100 suppressed AngII-induced YAP/TAZ nuclear translocation, the protein expressions of YAP **(D)** and TAZ **(F)** in the nucleus fraction, and the protein expressions of p-YAP **(E)** and p-TAZ **(G)** in the cytosolic fraction of HUVECs; protein expressions of proinflammatory cytokines MCP-1 **(H)** and TNFα. **(I)** *p < 0.05, vs. Ctr group, ^#^*p* < 0.05, vs. AngII group.

Consistent with the findings *in vivo*, AngII significantly increased the protein expressions of YAP and TAZ in the nucleus fraction (Figures 5A,C) associated with reduced expressions of p-YAP and p-TAZ in the cytosolic fraction of HUVECs *in vitro* (Figures 5B,D), and immunofluorescence staining showed that AngII increased YAP immunofluorescence intensity in the nucleus of HUEVCs. Treatment with verteporfin reduced AngII-induced nucleus relocation of YAP (Figures 5E,F). Consistent with the findings *in vivo*, the inhibition of YAP/TAZ with verteporfin in HUVECs *in vitro* prevented a decrease in AngII-induced p-eNOS expression (Figure 6A) and an increase in the expressions of proinflammatory cytokines MCP-1 and TNFα (Figures 6B,C). These results suggest that AngII can activate YAP or TAZ in the endothelium, which may contribute to AngII-induced endothelial activation. PP2A is a multifunctional serine/threonine phosphatase. Phosphorylated PP2A is an inactive form, and after dephosphorylation, PP2A is activated. PP2Ac is a major catalytic subunit, and it has been shown that MST1/2 kinases are dephosphorylated by PP2Ac, thus inhibiting the Hippo pathway and activating YAP (Hata et al., 2013). We determined the PP2Ac activity in HUVECs, using a V2460 kit from Promega (Madison, WI, United States) (Ding et al., 2020). As shown in Figure 7A, AngII significantly increased PP2Ac activity, and treatment with PP2Ac inhibitor LB-100 reduced PP2Ac activity in AngII-treated cells. Phosphor-PP2Ac (an inactive form of PP2Ac) but no PP2Ac in AngII-treated cells was significantly decreased, which was significantly reversed in the LB-100-treated group (Figures 7B,C). To investigate whether PP2Ac is involved in AngII-induced YAP activation in the endothelium, HUEVCs were incubated with AngII with or without LB-100. As shown in Figures 7D–G, the inhibition of PP2Ac with LB-100 prevented a decrease in AngII-induced cytoplasmic YAP/TAZ phosphorylation at ser127 and an increase in nucleus YAP/TAZ expression, suggesting that AngII activates YAP/TAZ signaling *via* the stimulation of PP2Ac. To further investigate whether PP2Ac activation of YAP/TAZ participates in the regulation of AngII-induced endothelial activation, we determined the protein expressions of proinflammatory cytokines TNFα and MCP-1 in AngII-treated HUVECs. AngII significantly increased the expressions of TNFα and MCP-1, and treatment with LB-100 reversed the expressions of AngII-induced proinflammatory cytokines (Figures 7H,I).

## Discussion

This study demonstrates that vascular YAP/TAZ are activated in AngII hypertensive mice. The inhibition of YAP/TAZ with verteporfin improves endothelial function, vascular inflammation, and fibrosis associated with a mild reduction in arterial blood pressure. In cultured HUVECs, AngII activates the YAP/TAZ pathway and induces endothelial activation *via* the stimulation of PP2Ac dephosphorylation. These results suggest that the activation of endothelial YAP/TAZ plays an important role in endothelial dysfunction, vascular inflammation, and fibrosis in AngII hypertension.

Yes-associated protein/TAZ is a highly conserved molecular component and downstream effector of the Hippo pathway. YAP and TAZ are transcriptional co-regulators, YAP does not have intrinsic DNA-binding domains, and YAP needs cooperation with TAZ to bind the promoters of target genes to regulate the transcription of the target genes (Pocaterra et al., 2020). The Hippo signaling pathway induces the phosphorylation of YAP/TAZ at multiple serine residues, causing them inactivation and retention in the cytoplasm. The dephosphorylation of YAP/TAZ promotes their nuclear translocation and regulates the expressions of their target genes (Zheng and Pan, 2019). The infusion of AngII decreases phosphorylated YAP/TAZ in the cytosolic fraction and increases the nuclear relocation of YAP/TAZ in the aorta of hypertensive mice, which are prevented by treatment with verteporfin. These results suggest that YAP/TAZ can be activated in the vascular system of AngII hypertension. YAP/TAZ is sensitive to biomechanical stress including hemodynamic stress, particularly disturbed blood flow (Wang L. et al., 2016). It has been shown that a turbulent flow can activate YAP/TAZ in HUVECs (Wang K.C. et al., 2016). High blood pressure often causes a turbulent blood flow in the vascular system, which may induce YAP/TAZ activation in the vascular cells, particularly in endothelial cells. Endothelial cells directly contact and sensitize to blood flow and hemodynamic stress. Since AngII can directly activate YAP/TAZ in HUVECs *in vitro*, it is difficult to determine to what extent activation of vascular YAP/TAZ is AngII-dependent or blood pressure-dependent in this animal model. When the Hippo pathway is inactivated, the YAP/TAZ is usually dephosphorylated by LATS1/2 on serine residue, which leads to their translocation into the nucleus and activation (Ma et al., 2019). PP2Ac is a highly conserved serine/threonine dephosphorylation protein. It has been shown that AngII activates PP2Ac, which induces eNOS dephosphorylation at ser 1179 and causes endothelial dysfunction (Ding et al., 2020). This study indicates that AngII can activate PP2Ac, and the inhibition of PP2Ac with LB-100 suppresses the AngII activation of YAP/TAZ in HUVECs, suggesting that AngII induces YAP/TAZ activation *via* PP2Ac-mediated YAP/TAZ dephosphorylation.

Excess activation of the renin-angiotensin system plays an essential role in the pathogenesis of hypertension. AngII is a proinflammatory mediator, which increases endothelial expressions of MCP-1, vascular cell-adhesive molecule 1 (VCAM1), and intracellular adhesive molecule 1 and enhances macrophage infiltration into the vascular wall. The recruitment of macrophages in the vascular wall is critical for the initiation and maintenance of vascular inflammation in hypertension. Recent studies have shown that the Hippo/YAP pathway may participate in the regulation of vascular inflammation. Wang et al. have demonstrated that laminar shear stress suppresses endothelial inflammation and macrophage recruitment in the vascular wall *via* the inhibition of YAP/TAZ activation (Wang L. et al., 2016). In contrast, disturbed blood flow activates the endothelial YAP/TAZ pathway, therefore promotes endothelial inflammation and atherogenesis (Wang L. et al., 2016; Xu et al., 2016). Choi et al. (2018) reported that knockdown of YAP/TAZ genes reduces TNFα-induced VCAM1 expression. In this study, we demonstrated that the inhibition of YAP/TAZ by verteporfin reduces the expression of proinflammatory cytokines MCP-1 and TNFα *in vivo* and *in vitro* and macrophage infiltration *in vivo*. MCP-1 is an important chemokine to mediate macrophage infiltration. Infiltrated macrophage releases inflammatory cytokines, such as TNFα and interleukin-1β, which may initiate endothelial dysfunction, oxidative stress, vascular inflammation, and fibrosis (Remus et al., 2015; Konukoglu and Uzun, 2017). Therefore, we surmised that the AngII activation of the YAP/TAZ pathway may cause vascular inflammation and endothelial dysfunction in hypertension.

It should be noted that the effects of AngII on the vascular system and its target organs are very complex. AngII mainly mediates its biological effects *via* angiotensin type 1 receptor (AT1R). The activation of AT1R can promote and interact with various intracellular signaling pathways, resulting in endothelial dysfunction, vascular inflammation, and remodeling (Chen et al., 2020). In addition, AngII can also exert an indirect effect on the cardiovascular system *via* the central nervous system or hemodynamics (Peters et al., 2015; Farag et al., 2017). We have shown that AngII can directly activate the YAP/TAZ pathway in endothelium *in vitro*. Since hemodynamic stress plays a crucial role in the activation of the YAP/TAZ pathway, it cannot be excluded that the AngII activation of YAP/TAZ *in vivo* is partly secondary to its hemodynamic effects.

### Limitations

This study has several limitations. First, we may miss a group in normal mice with verteporfin treatment, and although the results of this group may not affect our conclusion, it may help us understand the vascular effects of verteporfin on normal mice. Then, hypertension and AngII can affect different vascular beds, resistance artery plays an important role in the regulation of vascular tone and blood pressure, and this study only analyzes verteporfin effects on aorta but not resistance artery. Finally, it has been shown that T lymphocytes and different macrophage subsets play the role in hypertensive vascular injury (Guzik et al., 2007; Carnevale et al., 2020), we did not detect the markers of T cells and macrophage subsets in the vascular system, and it is unclear whether the activation of the YAP/TAZ pathway involves these immune cells in AngII hypertensive mice, which is worthy to be investigated in future.

### Conclusion

This study demonstrates that the infusion of AngII activates the endothelial YAP/TAZ pathway, which importantly contributes to vascular inflammation and endothelial dysfunction in hypertensive mice. AngII may activate YAP/TAZ by PP2Ac-dependent dephosphorylation of YAP/TAZ. Our results suggest that YAP/TAZ play a critical role in hypertensive vascular injury and may be a new target molecule for hypertension.

## Data Availability Statement

The original contributions presented in the study are included in the article/supplementary material, further inquiries can be directed to the corresponding author/s.

## Ethics Statement

The animal study was reviewed and approved by Institutional Animal Care and Use Committee of Shenyang Medical College.

## Author Contributions

QX contributed to the conception and design of this study, acquisition of data, analysis, interpretation of data, and statistical analysis. KZ contributed to the acquisition of data, analysis, interpretation of data, and statistical analysis. RC, XS, LuZ, YL, LiZ, and FR contributed to the acquisition of data, analysis, and interpretation of data. M-SZ contributed to the conception and design of this study, analysis, interpretation of data, and drafted the manuscript. All authors contributed to the article and approved the submitted version.

## Conflict of Interest

The authors declare that the research was conducted in the absence of any commercial or financial relationships that could be construed as a potential conflict of interest.

## Publisher’s Note

All claims expressed in this article are solely those of the authors and do not necessarily represent those of their affiliated organizations, or those of the publisher, the editors and the reviewers. Any product that may be evaluated in this article, or claim that may be made by its manufacturer, is not guaranteed or endorsed by the publisher.
